# The Factors Associated with the Blood–Brain Barrier Dysfunction in Tick-Borne Encephalitis

**DOI:** 10.3390/ijms26041503

**Published:** 2025-02-11

**Authors:** Sambor Grygorczuk, Piotr Czupryna, Diana Martonik, Justyna Adamczuk, Anna Parfieniuk-Kowerda, Anna Grzeszczuk, Wioletta Pawlak-Zalewska, Justyna Dunaj-Małyszko, Kaja Mielczak, Miłosz Parczewski, Anna Moniuszko-Malinowska

**Affiliations:** 1 Department of Infectious Diseases and Neuroinfections, Medical University in Białystok, 15-089 Białystok, Poland; piotr.czupryna@umb.edu.pl (P.C.); justyna.adamczuk@umb.edu.pl (J.A.); anna.grzeszczuk@umb.edu.pl (A.G.); wioletta.pawlak-zalewska@umb.edu.pl (W.P.-Z.); justyna.dunaj@umb.edu.pl (J.D.-M.); anna.moniuszko@umb.edu.pl (A.M.-M.); 2 Department of Infectious Diseases and Hepatology, Medical University in Białystok, 15-089 Białystok, Poland; diana.martonik@umb.edu.pl (D.M.); anna.parfieniuk@umb.edu.pl (A.P.-K.); 3 Department of Infectious, Tropical Diseases and Acquired Immunodeficiency, Pomeranian Medical University, 70-204 Szczecin, Poland; kmielczak@pum.edu.pl (K.M.); mparczewski@yahoo.co.uk (M.P.)

**Keywords:** *Flavivirus* encephalitis, tick-borne encephalitis, blood–brain barrier, neuroinflammation

## Abstract

The pathogenesis of the central nervous system (CNS) pathology in tick-borne encephalitis (TBE) remains unclear. We attempted to identify mediators of the blood–brain barrier (BBB) disruption in human TBE in paired serum and cerebrospinal fluid (CSF) samples from 100 TBE patients. CSF albumin quotient (Q_alb_) was calculated as a measure of BBB impairment. Concentrations of cytokines, cytokine antagonists, adhesion molecules, selectins and matrix metalloproteinases (MMP) were measured with a multiplex bead assay. Single nucleotide polymorphisms (SNP) in genes *MIF*, *TNF*, *TNFRSF1A*, *TNFRSF1B*, *IL-10*, *TLR3* and *TLR4* were studied in patient blood DNA extracts and analyzed for associations with Q_alb_ and/or cytokine concentrations. The multivariate regression models of Q_alb_ were built with the soluble mediators as independent variables. The best models obtained included L-selectin, P-selectin, sVCAM, MMP7, MMP8 (or MMP9) and IL-28A as positive and IL-12p70, IL-15, IL-6Rα/IL-6 ratio and TNF-RII/TNFα ratio as negative correlates of Q_alb_. The genotype did not associate with Q_alb_, but polymorphism rs4149570 (in *TNFRSF1A*) associated with TNFα and rs1800629 (*TNF*) with MIF concentration. We confirm the association of the TNFα-dependent response, L-selectin and MMP8/MMP9 with BBB disruption and identify its novel correlates (IL-12, IL-15, IL-28A, MMP7). We detect no genotype associations with BBB function in TBE.

## 1. Introduction

The blood–brain barrier (BBB) consists of the functional and anatomical barriers preventing, in normal conditions, the leakage of macromolecular compounds and cells between the circulating blood and central nervous system (CNS). It is formed by the brain endothelial basement membrane and a layer of brain endothelial cells (BECs) connected by impermeable tight junctions [1,2,3]. In a wider sense it also includes the glial barrier surrounding the perivascular space, permeable to soluble substances but further restricting the migration of extravasated immune cells into the brain parenchyma [3,4,5]. The BBB is able to transduce neuroimmune and inflammatory signals between the vascular and CNS compartments without losing functional and anatomical integrity [6]. In inflammation, however, BBB disruption may allow for an uncontrolled influx of macromolecular compounds, immune cells and microorganisms into the CNS, with potential pathogenic consequences [1,6,7].

Tick-borne encephalitis (TBE) is caused by the TBE virus (TBEV) present in a natural reservoir and causes human infections in a large area stretching from Central Europe to the Far East of Asia [8,9]. In 2022, 20 European Union/European Economic Area (EU/EEA) countries reported a total of 3650 cases of tick-borne encephalitis [9]. In 2023, 659 cases were reported in Poland alone (morbidity 1.75/100,000 population), of which 213 were concentrated in the highly endemic area in the Podlaskie province in the north-east of the country (local morbidity 18.67/100,000) [10]. The disruption of the BBB is a hallmark of the *Flavivirus* encephalitis, including TBE as well as West Nile encephalitis (WNE) and Japanese encephalitis (JE) caused by related mosquito-borne flaviviruses. BBB impairment was described in animal models of encephalitis [11,12] and is reflected by an increased cerebrospinal fluid (CSF) albumin concentration in patients in the clinical setting [13,14]. There is no evident association between the BBB disruption and the clinical severity of human TBE [13]. However, the mechanism of the CNS injury in TBE is not fully understood and it may depend to a larger extent on the inflammation and immune-mediated pathology than on a detectable viral burden [15,16]. The initial *Flavivirus* entry into the CNS may occur through an intact BBB, with the virus entering BECs luminally from the circulating blood, replicating in them and being released abluminally into the CNS [7,11,17,18]. However, as shown by Daniels et al., West Nile virus (WNV) penetration across the BEC layer is facilitated by a disruption of intracellular junctions induced by circulating proinflammatory cytokines [12]. The signaling axis initiated by toll-like receptors TLR3 and TLR4, mediated by an up-stream proinflammatory cytokine, macrophage migration inhibitory factor (MIF), effector cytokines TNFα, IL-1β and/or IL-6, and modulated by IL-10, was proposed a main route of the BBB impairment in animal models of *Flavivirus* encephalitis [12,19,20,21]. On the other hand, the effective peripheral type I interferon response, dependent on the TLR7 activation, has been proposed to decrease the BBB disruption by preventing excessive inflammation in the periphery [12].

Proinflammatory cytokines may directly influence the transcription of the proteins important for BEC layer permeability, for example TNFα abrogates claudin-2 expression and IL-1β downregulates expression of occludin [22,23], but several additional downstream mediators may be involved in a BBB disruption on the effector level. Vascular endothelial growth factor (VEGF), synthesized by astrocytes in intrathecal inflammation, increases BBB permeability by downregulating the expression of tight junction proteins claudin-5 and occludin, which directly influences BBB structure and function [22,24,25]. Matrix metalloproteinases (MMPs) may contribute to BBB damage by lysing its constituent proteins [4,5,25], and are upregulated by proinflammatory cytokines in vitro and in infected neuron or astrocyte cultures [25,26,27,28] and in viral CNS infections in vivo [4,29,30,31,32,33].

**Figure 1:**
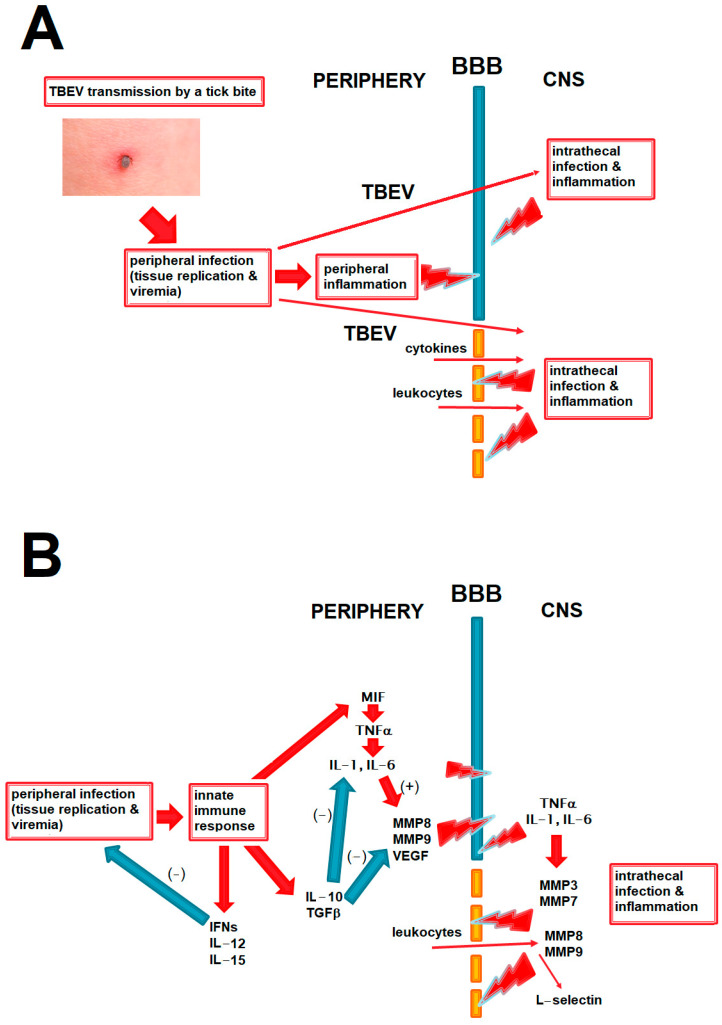
The diagrams summing up the pathogenetic pathways relevant to BBB disruption in TBE and discussed in the paper. The red arrows signify a positive and the blue arrows a negative (inhibitory) effect. (**A**) The main elements in the pathogenesis of the CNS infection and inflammation in TBE. TBE virus circulating in periphery can enter the CNS through intact BBB, but an inflammatory disruption of the BBB may further intensify this process. BBB disruption may be initiated by inflammatory mediators acting from the luminal side during peripheral infection and further exacerbated by a following inflammation within the CNS in the neurologic phase of the disease. BBB disruption may further contribute to CNS inflammation by facilitating access of inflammatory mediators and cells from periphery. Positive feedback loops between these elements are likely, but their exact interrelation in a human disease is not known. (**B**) The hypothesized place of the main groups of mediators analyzed in the current study in the pathogenesis of neuroinflammation and BBB disruption in TBE. Proinflammatory cytokines may disrupt the BBB directly and through upregulating matrix metalloproteinases (MMPs), acting both from luminal side in peripheral inflammation and abluminally during CNS infections; this may be attenuated by anti-inflammatory and immunomodulatory cytokines like IL-10. Effective antiviral response in periphery, for example mediated by interferons of IL-15, may protect the BBB by reducing peripheral viral burden and inflammation. MMPs act on the BBB from the luminal side (e.g., MMP8 and MMP9 from leukocytes) and abluminal side (derived from infiltrating leukocytes and/or from glial cells). L-selectin may be upregulated intrathecally as a result of leukocyte infiltration, being rather a marker than a cause of the BBB disruption. BBB—blood–brain barrier, CNS—central nervous system, IFNs—interferons, TGF—transforming growth factor, TNF—tumor necrosis factor, MMPs—matrix metalloproteinases, VEGF—vascular endothelial growth factor, TBE—tick-borne encephalitis.

We present the potential role of BBB disruption in TBE in relation to peripheral and intrathecal inflammation and viral spread into the CNS in Figure 1A and the hypothesized role of the main cytokines and other mediators discussed in this study in that process in Figure 1B.

To clarify the pathogenesis of BBB impairment in human TBE, we have studied the expression of multiple mediators potentially contributing to it and analyzed their associations with the CSF albumin quotient as a biochemical marker of the BBB function, in TBE patients.

## 2. Results

### 2.1. Study Group

The main study cohort consisted of 100 TBE patients, 64 male and 36 female, aged 19 to 79 years (median 48.0 y), 49 with the disease presenting as meningitis (M), 46 with meningoencephalitis (ME) and 5 with meningoencephalomyelitis (MEM). ME and MEM patients tended to be older than those with M (median age of 52.5, 52.0 and 44.0 years, respectively, *p* = 0.085). There were 20 patients with mild, 76 with moderately severe and 4 with severe TBE according to the Bogovič scale [34] (Appendix D, Figure A5). There were no pregnant women, patients with severe immunodeficiency or undergoing immunosuppressive therapy or patients with a probable other etiology of the CNS infection. Three TBE patients had a history of anti-TBE vaccination (uncomplete in two cases), but the clinical presentation and the study results in them did not differ systematically from the rest of the cohort. Because of the limited volumes of serum and CSF available, concentrations of some mediators were not measured in individual samples. For most of the mediators, the data are available for 94–98 serum and 95–100 CSF samples, with an exception of IL-22, which was studied in 88 CSF samples only.

The same mediators as in the main study cohort were assessed in 19 control serum samples from healthy volunteers and 11 control (non-inflammatory) CSF samples from patients without neuroinflammation.

The analysis of the associations of the mediator concentrations with the genetic polymorphisms was performed on data from 89 patients from the above group and the analysis of Q_alb_ genetic associations—on a larger group, including 94 patients from the main study cohort supplemented by retrospective data from 120 patients studied in the previous genotyping research program during 2016–2018. The additional patients did not differ significantly from the main cohort in terms of demographics and clinical presentation.

### 2.2. Blood–Brain Barrier Function

Table 1 shows the CSF cellular parameters, albumin concentration and Q_alb_ values in TBE patients stratified according to clinical presentation and severity. Q_alb_ was elevated in 82 (82%) patients. The trend for moderately higher albumin concentration and Q_alb_ in MEM and in the most severely ill patients was not significant. A weak trend for a positive correlation between Q_alb_ and a numerical value of a Bogovič score was non-significant, too (Table 1).

Q_alb_ correlated positively with total leukocyte and lymphocyte counts, but not with a neutrophil count, suggesting that it was associated with a more mature and/or specific immune response (Table 2).

### 2.3. Concentrations of the Soluble Mediators

The basic results for all the soluble mediators included in the multivariate regression analyses, including their median concentrations in serum and CSF, their comparison to values in non-inflammatory control samples and their correlation with Q_alb_, are listed in Table 3.

#### 2.3.1. Proinflammatory Cytokines and Their Soluble Antagonists

**Figure 2:**
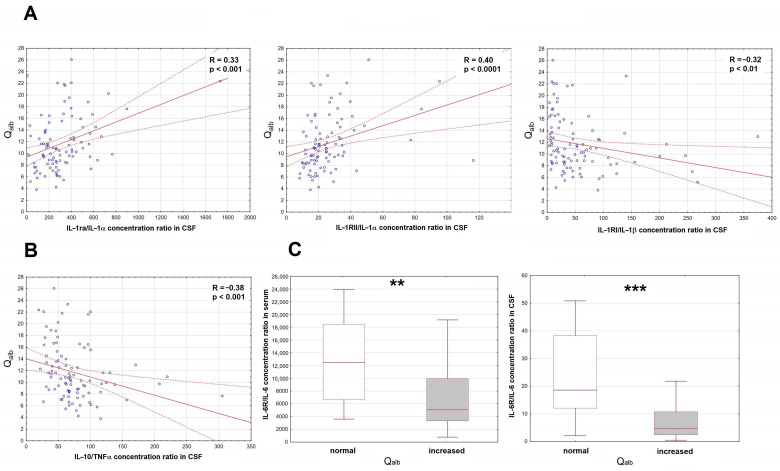
The associations of the cerebrospinal fluid albumin quotient (Q_alb_) with the ratios of the correlations of the soluble antagonists and the proinflammatory cytokines in patients with tick-borne encephalitis. (**A**) The scatter plots presenting the positive correlation of the IL-1ra/IL-1α ratio (**left panel**, n = 95), IL-1RII/IL-1α ratio (**middle panel**, n = 95) and negative correlation of the IL-1RI/IL-1β ratio (**right panel**, n = 99) in cerebrospinal fluid (CSF), shown on the horizontal axis, with Q_alb_ shown on vertical axis. The individual data points are shown with blue circles, the best linear fit with a continuous red line and the 95% confidence interval with red dashed lines. Two extreme cases were omitted from the plot in the right panel, but not from the analysis. The strength of the correlation and the statistical significance are presented on the plots. (**B**) The scatter plot showing the negative linear correlation between the IL-10/TNFα concentration ratio in CSF (on vertical axis) with Q_alb_ (on horizontal axis), n = 95. The individual data points are shown with blue circles, the best linear fit with a continuous red line and the 95% confidence interval with red dashed lines. The strength and statistical significance of the correlation are presented on the plot. (**C**) The comparison of the IL-6Rα/IL-6 concentration ratios in serum (**left panel**) and CSF (**right panel**) between the patients with the normal (n = 18) and increased (n = 78 for serum, n = 80 for CSF) Q_alb_ value. Shown are the median (horizontal line), quartile range (box) and non-extreme value range (whiskers). Six extreme data points (extremely high ratios) were omitted from the left and five from the right plot for clarity, but were included in the analysis. **—statistically significant difference between the groups with *p* < 0.01; ***—with *p* < 0.001.

Of the initial set of the proinflammatory factors, only the IL-6 concentration was increased in serum. Of the antagonistic/anti-inflammatory factors, there were increased concentrations of sTNF-RI, IL-1ra and IL-10. In CSF, concentrations of MIF, TNFα, IL-1α, IL-1β, IL-6 and IL-12 were all increased and correlated with Q_alb_, but the same trends were found for most of their potential antagonists. To disentangle their roles, we have analyzed the ratios of the potential antagonists to the concentrations of the respective proinflammatory factors (IL-1ra, sIL-1RI and sIL-1RII to IL-1α and IL-1β, sTNFRI and sTNFRII to TNFα, sIL-6Rα to IL-6, IL-10 to TNFα). As expected, the IL-10/TNFα and sIL-1RI/IL-1β ratios correlated inversely with Q_alb_, but for IL-1RII/IL-1α and IL-1ra/IL-1α, the correlation was surprisingly opposite (Figure 2A,B). Both serum and CSF ratios of IL-6Rα/IL-6 tended to correlate negatively with Q_alb_, which was most evident when we compared IL-6Rα/IL-6 values between the subgroups of patients with normal (n = 18) and elevated (n = 82) Q_alb_ (Figure 2C).

#### 2.3.2. Matrix Metalloproteinases

**Figure 3:**
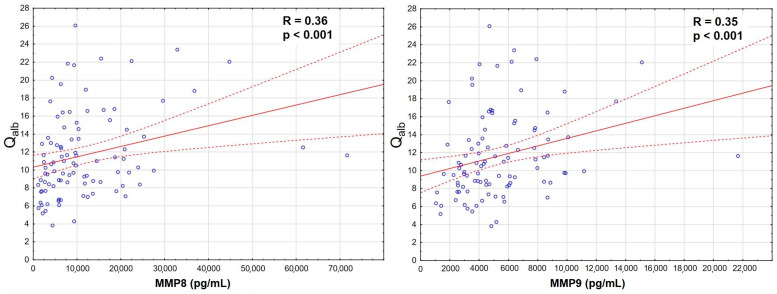
The positive correlation of the cerebrospinal fluid albumin quotient (Q_alb_, vertical axis) with the serum concentrations of matrix metalloproteinases (horizontal axis): MMP8 (**left panel**, in pg/mL, n = 96) and MMP9 (**right panel**, in ng/mL, n = 98) in sera of patients with tick-borne encephalitis. The individual data points are shown with blue circles, the best linear fit with a continuous red line and the 95% confidence interval with dashed red lines. The strength and statistical significance of the correlation are presented directly on the plots.

**Figure A1:**
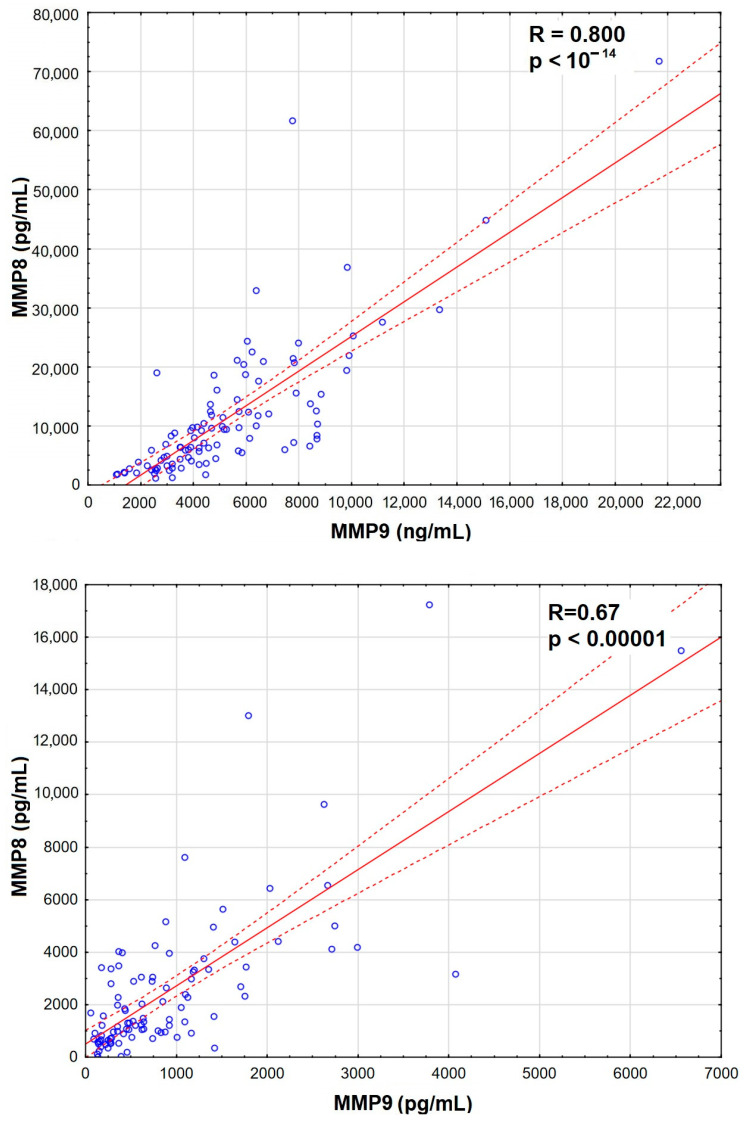
A scatter plot presenting the strong and very highly significantly positive correlation between the concentrations of matrix metalloproteinases MMP8 (vertical axis, expressed in pg/mL) and MMP9 (horizontal axis, expressed in ng/mL) in sera (**top**) and CSF (**bottom**) of 96 patients with tick-borne encephalitis. The individual data points are shown with blue circles, the best linear fit with a red continuous line and the 95% confidence interval with red dashed lines. The strength and statistical significance of the correlation are presented directly on the plot.

The increase was the most significant for MMP8 and MMP9 in serum, which also correlated strongly with Q_alb_ (Figure 3), while MMP7 and MMP12 were actually downregulated in TBE. There was a prominent correlation between MMP8 and MMP9 concentrations in serum, stronger than their individual correlations with Q_alb_, and a slightly weaker correlation in CSF (Appendix A, Figure A1).

We attempted to create a multivariate regression model of Q_alb_ including the MIF signaling axis cytokines, their antagonists and MMPs. The models most robustly associated with Q_alb_ included MIF, TNFα and sIL-1RI concentrations in CSF as well as either MMP8 or MMP9 concentrations in serum, with marginally better parameters for the model including MMP8 versus MMP9 (Appendix B, Table A1). The model predicted the association of the most vividly upregulated MMPs and of TNFα with Q_alb_, although only CSF and not serum TNFα concentration was recovered. Moreover, MIF and sIL-1RI associated with Q_alb_ with different signs than expected and the overall R^2^ value and statistical significance were low, suggesting the need of introducing additional variables.

#### 2.3.3. VEGF, Adhesion Molecules and Selectins

Most of the factors in this group were upregulated intrathecally and their CSF concentrations correlated highly significantly with Q_alb_. The increase was especially vivid for sVCAM-1 and L-selectin CSF concentrations. VEGF concentration was only modestly increased and its correlation with Q_alb_ relatively weak.

#### 2.3.4. Additional Cytokines and Final Multivariate Regression Analysis

We evaluated concentrations of the additional inflammatory and antiviral cytokines available from the same sample set—G-CSF, GM-CSF, IFNα_1_, IFNα_2_, IFNβ, IFNγ, IL-2, IL-15, IL-16, IL-17A, IL-17E, IL-17F, IL-18, IL-21, IL-22, IL-28A, IL-28B, IL-29 and IL-33—with the rationale that they may be able to influence the extent of the peripheral and intrathecal inflammation either directly or by their antiviral effects, and thus contribute to the BBB status. Of these mediators, IFNα_2_, IFNβ, IL-29, IL-17A, IL-17E and IL-21 were neither significantly up-regulated nor correlated with Q_alb_ in the study cohort and were not analyzed further. The CSF and, in selected cases, serum concentrations of the remaining cytokines were tested in the multivariate regression analyses.

**Figure 4:**
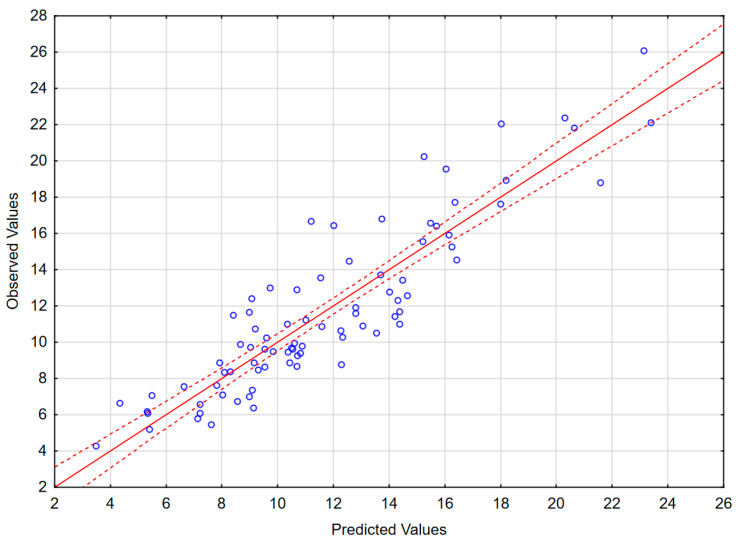
The scatter plot of the observed values of the Q_alb_ in a study population of TBE patients (vertical axis) versus the predicted values calculated with a model presented in Table 4 (horizontal axis) (n = 83). The individual data points are shown with blue circles, the model linear function with a continuous red line and the 95% confidence interval with dashed red lines.

**Figure A2:**
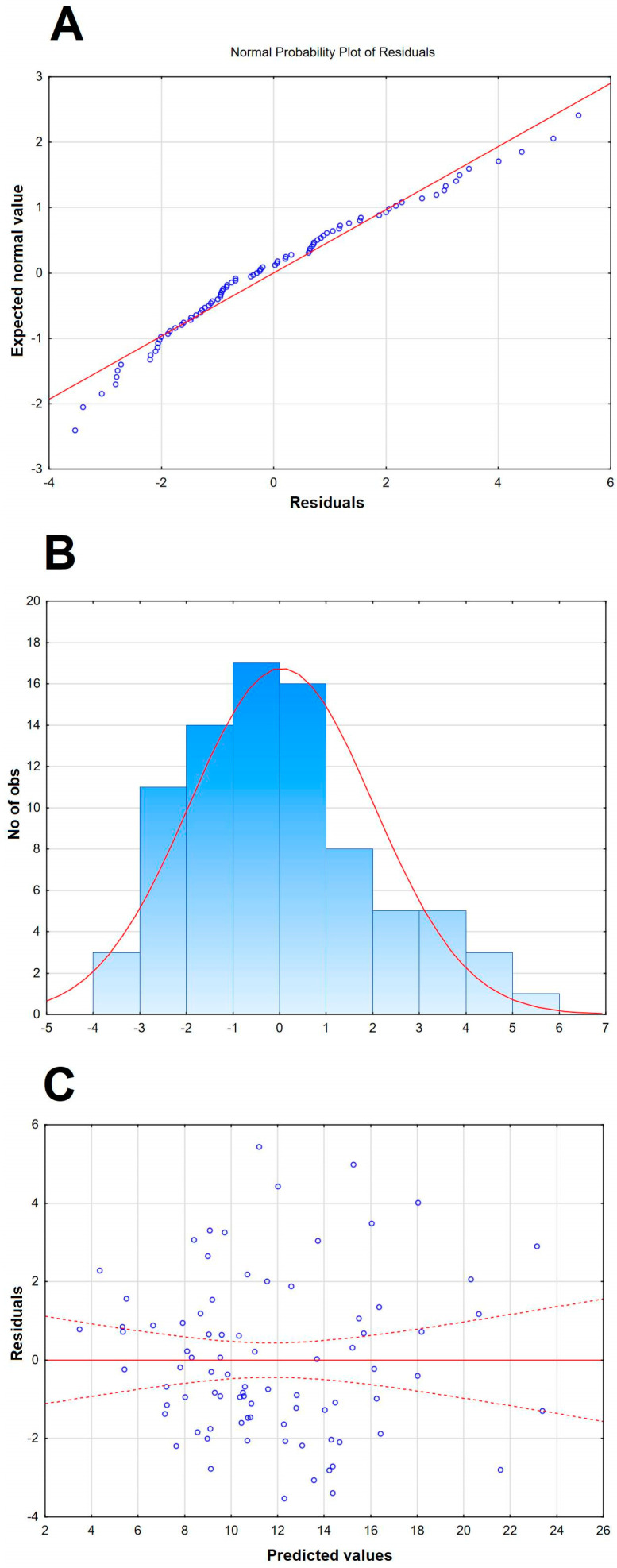
A graphic illustration of the selected additional features of the Q_alb_ model presented in Table 4 and in Figure 3. (**A**) The normal probability plot of residuals, with individual cases presented with circles, showing a good fit to normal. (**B**) The histogram of the distribution of raw residuals, with a best fit Gauss curve shown with a red line and the number of observed cases in each numerical bin described on the left vertical axis. (**C**) The scatter plot of the residuals versus predicted Q_alb_ values, with individual values presented with blue circles, showing the even distribution of residuals.

**Figure A3:**
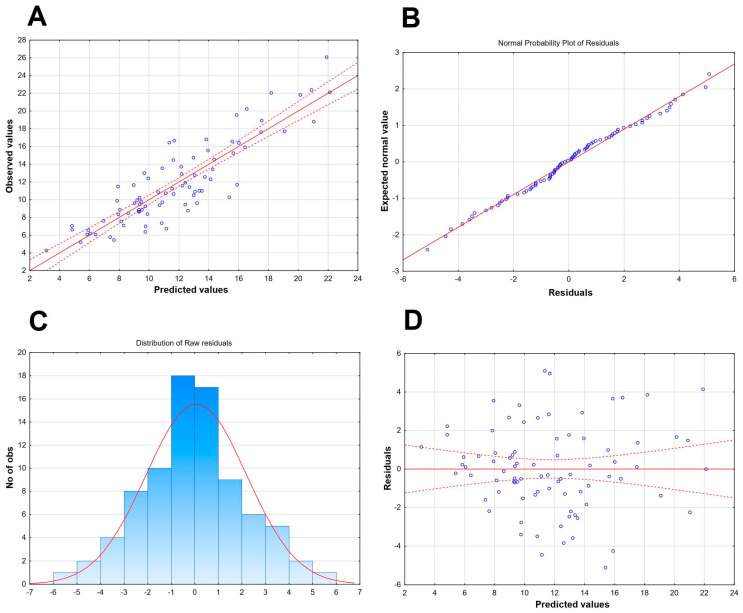
A graphic illustration of the selected features of the alternative Q_alb_ model presented in Table A2. (**A**) The scatter plot of the observed values of the Q_alb_ in a study population of TBE patients (vertical axis) versus the predicted values calculated with the model. The individual data points are shown with blue circles, the model function with a continuous red line and the 95% confidence interval with red dashed lines. (**B**) The normal probability plot of residuals, with individual cases presented with circles, showing a good fit to normal (red line). (**C**) The histogram of the distribution of raw residuals, with a best fit Gauss curve presented with a red line and the number of observed cases in each numerical bin described on the left vertical axis. (**D**) The scatter plot of the residuals versus predicted Q_alb_ values, with individual values presented with circles, showing the even distribution of residuals.

The results of the best obtained model are presented in Table 4, Figure 4 and in Appendix B (Figure A2). The model includes 11 independent variables: L-selectin in CSF, P-selectin in CSF, sVCAM in CSF, MMP8 in serum, MMP7 in CSF, IL-12p70 in serum, IL-15 in serum, TNF-RII to TNFα ratio in CSF, IL-6Rα to IL-6 ratio in serum, IL-28A in serum and MIF in serum, as well as a realistic and highly statistically significant free parameter. MIF does not correlate significantly with Q_alb_, but is retained because its exclusion deteriorates the model parameters. In spite of the large number of independent variables, the intercorrelation level between them was acceptable (tolerance coefficient > 0.59 for all the variables, partial correlation coefficient higher than the R-square value for all except MIF, for which these parameters were both low, 0.08 and 0.13, respectively). The inclusion of MMP9 instead of MMP8 led to a similar model with only marginally lower F and R^2^ values. The moderately weaker, but consistent model not including TNF-RII/TNFα and IL-6Rα/IL-6 ratios but including IFNγ as a significant independent value is shown in Appendix B (Table A2 and Figure A3). The feasible models could also be created involving (1) MMP3 in serum instead of MMP7 in CSF as a positive predictive factor; (2) IL-2, IL-16 and/or IFNα_1_ in serum, the latest in spite of being detectable only in a minority of sera, instead of IL-12 and/or IL-15 as negative predictive factors; (3) other changes, for example IL-15 in CSF instead of serum. However, all these models had moderately lower F or R^2^ values, weaker correlations with Q_alb_ for individual variables and/or an unrealistic free parameter. Other potentially pathogenetically significant variables, like VEGF, MMP1 or MMP2, were generally not recovered by the models.

### 2.4. The Genetic Analysis

The genotyping was successful in all the cases. The distribution of the studied genotypes in the whole analyzed TBE cohort and in the smaller group in which the soluble mediator concentrations were studied is shown in Appendix C (Table A3).

There were no significant differences in Q_alb_ between the TBE patients with different genotypes in any of the studied SNPs (Table A4).

**Figure 5:**
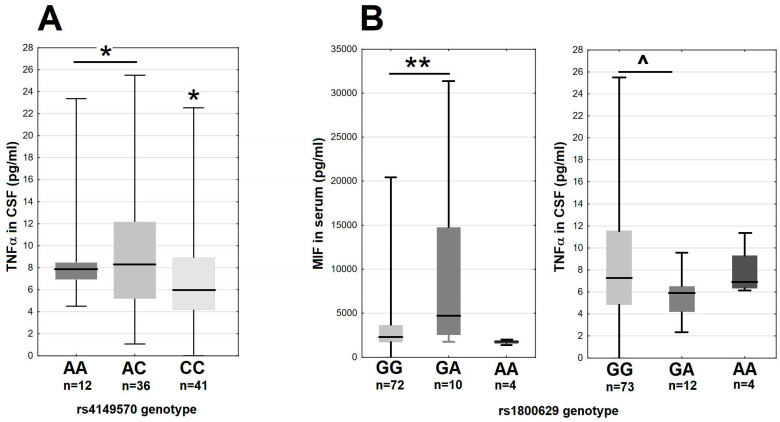
The TNFα and MIF expression in 89 tick-borne encephalitis patients on admission to hospital, dependent on the *TNFRSF1A* and *TNF* genotypes. (**A**) The TNFα concentration in cerebrospinal fluid stratified according to the rs4149570 genotype in *TNFRSF1A*. The median is shown by a horizontal line, quartiles by a box and the minimum–maximum range by whiskers. *—significant difference between the combined A/A–A/C genotypes and C/C genotype (*p* < 0.05 with Wilcoxon pair test and non-parametric ANOVA). (**B**) The MIF concentration in serum (**left**) and TNFα concentration in cerebrospinal fluid (**right**) stratified according to rs1800629 genotype in *TNF*. The median is shown by a horizontal line, quartiles by a box and the minimum–maximum range by whiskers. **—significant difference between GG and GA genotypes (*p* < 0.01 with Wilcoxon pair test); ^—the difference approaching the level of statistical significance with *p* = 0.062.

In the main study group, there were moderate differences of the MIF and TNFα expression associated with the variants of the genes coding the TNFα signaling axis. The minor A allele in rs4149570 locus in the gene for the TNFα receptor subunit *TNFRSF1A* associated with a 25% higher median TNFα level in CSF (Figure 5A). The GA heterozygotes in rs1800629 in *TNF* had over two-fold higher median MIF concentration in serum and a tendency for a lower TNFα concentration in CSF in comparison with the major G allele homozygotes (Figure 5B). The remaining SNPs in *TNF*, *TNFRSF1A* and *TNFRSF1B* did not associate with MIF, TNFα, sTNFRI or sTNFRII expression.

We found no other associations of the studied genotypes with the expression of relevant soluble factors. Especially, there was no association between (1) the *TLR3* and *TLR4* genotypes and the concentrations of the MIF-TNFα axis-related cytokines; (2) the *IL10* genotypes and Il-10 expression; and (3) *MIF* genotypes and CSF concentrations of MIF, TNFα, sTNFRI or sTNFRII.

## 3. Discussion

The most detailed data on the BBB function in *Flavivirus* encephalitis come from murine models, with a majority of studies focusing on infections with mosquito-borne WNV and JE virus (JEV), and with some discrepancies between different models and virus species [11,17,19,20,21,31]. The relation between CNS invasion by *Flavivirus*, leukocyte influx into the CNS and BBB disruption may differ between the models [12,17,18], leaving the pathogenesis of the human disease unclear. Several studies suggest the systemic inflammatory response in the early, peripheral phase of the infection causing initial BBB disruption, which facilitates virus entry into the CNS, resulting in encephalitis [19,20,21]. However, according to Roe et al., WNV may initially penetrate into the CNS through a physically intact BBB, only after which the CNS infection causes secondary BBB disruption, with a potential positive feedback between BBB impairment and neuroinflammation [18,31]. The analogous process of BBB disruption by the mediators (IL-6, VEGF and MMPs) released by infected astrocytes has been described in detail in an in vitro JEV infection model [25]. According to Daniels et al., both peripheral and intrathecal inflammation may affect BBB function in WNE sequentially. The authors documented BBB permeability increasing in two phases: first in the peripheral stage of the infection and then with the development of encephalitis [12]. As for TBE, in vitro and animal data suggest that TBEV initially enters the CNS via a transcellular pathway without affecting BBB structure and function and that BBB disruption is secondary to the established neuroinfection and neuroinflammation [11,17]. This difference puts in question the applicability of the results of the animal WNV and JEV studies regarding BBB function to human TBE and highlights the need for additional research on this topic.

In the current study, we have attempted to assess the contribution of different factors to BBB disruption in TBEV infection in humans. With that aim, we have analyzed correlations between the concentrations of soluble mediators and Q_alb_, chosen as a clinically available marker of BBB permeability to macromolecular compounds. We have studied multiple mediators of inflammation simultaneously, in a large patient cohort, to enable a detailed statistical analysis and testing of multiple hypotheses regarding TBE pathogenesis. As the CNS endothelial barrier may be sensitive to the mediators acting both from the vascular (luminal) and from the brain parenchyma (abluminal) side [6], we analyzed both serum and CSF concentrations. Interpretation of our results is limited by multiple intercorrelations between the studied mediator concentrations, as exemplified by a strong correlation between MMP8 and MMP9 in serum, but we were able to create feasible models while avoiding a simultaneous introduction of highly correlated independent variables. Our study captures the cytokine expression during the fully developed neurologic phase of TBE, simultaneously with the assessment of the BBB function. It is thus possible that we have underestimated the upregulation of proinflammatory factors expressed early in the course of the disease, during the peripheral infection, the initial BBB insult and the onset of the neurologic phase. Because of that, some of our negative results, for example regarding MIF expression in serum, may not be definitive.

The factors included in the study design were chosen on the basis of previous research and hypotheses on the pathogenesis of neurotropic *Flavivirus* infections and viral encephalitis in general. Our initial analysis included a set of proinflammatory cytokines related to TLR3-MIF-TNFα signaling axis, their soluble antagonists and potentially antagonistic cytokines (IL-10, TGFβ), selected based on the results of the murine WNE and JE studies [4,5,12,19,20,21]. In mice lacking expression of either toll-like receptor TLR3, the upstream mediator of inflammation MIF or the main receptor for TNFα–TNFR1, the presentation of WNE is mild, with reduced virus penetration across the BBB and inflammatory infiltrate within the CNS [19,20]. This may have suggested a signaling axis involving activation of TLR3, MIF as an upstream agonist and TNFα and proinflammatory cytokines upregulated by it (e.g., IL-1β, IL-6) to mediate BBB disruption in WNE [19,20]. The further studies confirmed these findings [4,5,12] and suggested that TNFα and IL-1β play most significant roles in murine WNE [12,19,35], while TNFα and IL-6 play most significant roles in murine JE [21]. IL-10 acts as an antagonist of TNFα-induced inflammation in a model of peripheral *Flavivirus* infection [15,21]. Consistently with animal model findings, in our previous study, we observed increased peripheral and intrathecal expression of MIF, TNFα and IL-1β and a correlation of serum MIF levels with CSF albumin concentration in TBE patients [14], while in a group of 78 patients with encephalitis of various etiology, described by Michael et al., higher concentrations of IL-10 in serum and CSF associated with a lower BBB permeability [36].

Our current results are consistent with the contribution of TNFα, IL-6 and IL-1β (but not IL-1α) and to BBB disruption. However, the effect of TNFα was detectable only for CSF and not serum concentration as would be expected for a peripheral inflammation initiating BBB breakdown and the role of MIF could not be confirmed. The best results were obtained when we analyzed concentration ratios of the proinflammatory cytokines and their soluble antagonists. This was expected, as cytokine/antagonist concentration ratios had already been shown to better associate with clinical features of viral encephalitis than cytokine concentrations per se [36]. Still, this set of mediators alone was insufficient to create a robust Q_alb_ model, strongly suggesting a complex, multifactorial pathogenesis of BBB disruption. As a result, we have included a number of additional independent variables in our further modeling attempts. Of these, the negative association of IL-15 with Q_alb_ is a novel finding as, to our knowledge, IL-15 has not been studied in viral encephalitis before, but it can be interpreted in a framework of the upregulated TNFα-dependent response. IL-15 is an IL-2-subfamily interleukin involved in a regulatory feedback network initiated by TNFα, its receptors are expressed on BECs and CNS parenchyma cells and it may modulate TNFα signaling in a complex way [23]. Although on the cellular level IL-15 is mostly proinflammatory, it abrogates some effects of TNFα and its overall activity in neuroinflammation may be protective. In experimental autoimmune encephalitis (EAE) in mice, IL-15 is upregulated and protective intrathecally and in a murine model of *neuromyelitis optica*, its expression in astrocytes decreases BBB permeability both for molecular compounds and for immune cells [37,38]. Accordingly, our models recovered IL-15 as associated with a better-preserved BBB function, with a correlation stronger for serum than for CSF concentration.

Another proinflammatory cytokine, IL-12, has been recovered as a factor associated with the milder BBB disruption, too. IL-12 is vividly expressed in TBE both in periphery and intrathecally and, alongside other Th1-related cytokines, is considered an element of the potentially harmful inflammatory response to TBEV [39,40]. In a group of 87 TBE patients described by Bogovič et al., concentrations of both subunits of IL-12 associated with a higher severity score [40]. However, our results suggest that the effect of IL-12 on BBB function may be more nuanced. Previously, Komatsu et al. showed in a murine model that the repeated injections of IL-12 not only did not cause a BBB pathology per se but also prevented the BBB disruption caused by an infection with vesicular stomatitis virus. According to the authors, this could reflect either the protective effect of IL-12 against viral infection or, more hypothetically, a result of a complex regulation of the nitric oxide synthetases activity at the BEC level by IL-12, modifying the response of the BBB to neuroinflammation [41]. Interestingly, the recent study by Marušić et al. suggests that both proinflammatory cytokines recovered as protective in our model, IL-12 and IL-15, may be important for the effective early response against TBEV, possibly as NK cell activators [42]. Our results suggest that their protective effects may extend into the neurologic phase of TBE.

It has been proposed that the peripheral type I IFN-dependent immune response may protect BBB integrity in an analogous manner, providing the early control of the *Flavivirus* infection and thus preventing an excessive TNFα-related inflammation [12,21,35]. For example, Welte et al. described a protective IFNα signaling dependent on TLR7 activation and unrelated to TNFα response in a peripheral phase of WNV murine infection [35]. In our TBE cohort, the concentrations of type I IFN were not increased in serum and, although a weak negative correlation with Q_alb_ could be recovered for IFNα_1_ in some modeling attempts, it was not recovered in the final model. On the other hand, we have observed a positive correlation of type II (IFNγ) and especially type III (IL-28A) IFN levels with Q_alb_. This is consistent with a TBE model described by Ružek et al., in which BBB dysfunction was temporally related to an increased expression of TNFα, IL-6 and IFNγ [11].

In summary, when analyzing cytokine concentration data, we find some consistency, but also a number of differences with the results of the murine models. The differences with the WNE and JE models include a Q_alb_ association with an intrathecal but not peripheral TNFα expression, a generally weak association with a MIF-TNFα–dependent response and a possibly different spectrum and role of interferons. These may reflect the discrepancy between animal models and a human infection or, more interestingly, the differences between the pathogenesis of TBE and of the, so far more intensively studied, infections with mosquito-borne flaviviruses. Our results are consistent with the BBB disruption in TBE being as a result of the primarily intrathecal instead of the peripheral inflammation, which agrees well with the results of TBEV in vitro and animal studies [11,17].

Several MMPs have been hypothesized to mediate BBB disruption in neuroinflammation. The BBB may be affected by MMPs expressed by both activated neutrophils and monocytes at the luminal side of the endothelium and by glial cells, especially astrocytes, at the parenchymal side [4]. The constitutive expression of mRNA for MMP3 and MMP12 was described in cultured astrocytes by Crocker et al. [27]. IL-1β upregulated MMP3 while downregulating MMP12 expression in this model [27], which agrees well with the changes of MMP3 and MMP12 concentrations in CSF in our study. Dawes et al. described an induced synthesis of MMP7 and MMP2 in a human neuron/astrocyte co-culture [26]. MMP, which is less consistently expressed by brain parenchyma cells [25,26,28], may be released by circulating or CNS-infiltrating neutrophils and monocytes [4,5,27]. The combined MMP synthesis by glia (MMP2, MMP3, MMP7) and leukocytes (MMP8, MMP9) may contribute to BBB disruption both on the astrocyte barrier and the endothelial basement membrane level. Especially MMP9, MMP8 and MMP3 are vividly upregulated intrathecally in murine encephalitis models, with a potential redundancy of their proteolytic effects [4,5]. Also, data on the varicella zoster virus (VZV) neurologic disease in humans suggest involvement of multiple MMPs, with an increased concentration of MMP2 and to a lesser degree of MMP3 and MMP9 both in serum and CSF, as well as of MMP8 and MMP12 in CSF only [32].

The data on the MMP expression in *Flavivirus* encephalitis suggest a more prominent role of MMP9, although MMP3 was also described as a factor causing BBB disruption in an animal model of WNE by Roe et al. [31]. In JE in mice, MMP9 is vividly upregulated intrathecally simultaneously with the development of the histopathological features of encephalitis, accompanied by MMP2 and MMP7 [30]. MMP9 concentration increases in WNV-infected mice in vivo in the order paralleling the progression of the disease: in serum simultaneously with viremia, in brain microvasculature before encephalitis onset and in the brain parenchyma in parallel with the progress of encephalitis. *MMP9* −/− mice have a reduced severity of encephalitis with a better-preserved BBB function [26]. MMP9 is secreted by astrocytes infected with a low virulence TBEV strain [28] and the increased concentration of MMP9 and increased ratio of MMP9 to its inhibitor, tissue inhibitor of metalloproteinase-1 (TIMP-1), were detected in sera of TBE patients [24]. In our dataset, it was impossible to distinguish the relative role of serum MMP8 and MMP9 because of their strong correlation and the final model includes MMP8. However, concentration of MMP9 was about an order of magnitude higher than of MMP8 and based on the literature data it is more likely to be the MMP determining the BBB disruption. The correlation was recovered only for serum MMP8 or MMP9 concentrations, suggesting that the main effect was exerted luminally. Unexpectedly, intrathecal MMP7 was recovered a second MMP affecting BBB integrity after peripheral MMP8 or MMP9. MMP7 was little studied in viral encephalitis before and to our knowledge has never been described as a pathogenetic factor in TBE before. However, it contributes to the intrathecal inflammation in EAE [43], is vividly upregulated in human neuron/astrocyte co-cultures infected with La Crosse virus [26] and has been detected in the CNS of JEV-infected mice [30]. Our early models also recovered MMP3, but the correlation was weaker and non-physiologically located in the periphery, while we do not find support for the pathogenetic role of MMP1, MMP2 or MMP12. In the future, the study of the MMP role could be refined by analyzing the ratios of their concentrations to their soluble inhibitors, tissue inhibitors of metalloproteinases (TIMPs), as they may be co-upregulated in neuroinflammation and the balance between them may determine the true level of the enzymatic activity of MMPs [24,26,44].

Of the soluble forms of the cell adhesion molecules, the association of the intrathecal L-selectin with BBB impairment was prominent, followed by P-selectin and sVCAM. Adhesion molecules and selectins were previously found to participate in the pathogenesis of viral meningitis and encephalitis. WNV infection causes increased expression of ICAM-1, VCAM-1 and E-selectin in endothelial monolayers in vitro, directly contributing to monocyte migration and resulting in BBB breakdown [31]. In children with enterovirus meningitis, there are increased serum and CSF levels of L-selectin, E-selectin and sICAM [42]. In a group of 37 patients with encephalitis of various aetiology, the CSF level of sVCAM, but not of sICAM, correlated strongly positively with Q_alb_, which is consistent with our observations [36]. L-selectin is expressed by leukocytes, involved in their migration across endothelia and shed on activation, rapidly by neutrophils and in a more gradual manner by lymphocytes—which suggests that it enables leukocyte migration into the CNS and is then released intrathecally [45]. While its membrane form may be involved in the pathogenesis of pleocytosis and the resulting intrathecal inflammation, a soluble form shed by leukocytes in CSF could be an end-product and a marker of this process. In a study by Buhrer et al., L-selectin was present in CSF of patients with encephalitis in concentrations implying its intrathecal release and correlating with a total protein concentration and leukocyte count and our data extend these observations by showing more directly its association with BBB impairment [46].

The role of some factors suspected to impact the BBB in encephalitis was not confirmed. Especially, a significant role of intrathecal VEGF in BBB disruption was proposed based on the murine autoimmune encephalitis model and in vitro experiments on human BECs [22]. In our study, VEGF concentration was moderately increased both peripherally and intrathecally where it correlated weakly with Q_alb_ in non-parametric tests, but it was not recovered by any multivariate regression models. Similarly, while TGFβ upregulates ocludin expression in vitro potentially improving BBB integrity [22], it did not present with any detectable protective effect in our study.

We have not identified any evident genetic correlates of the BBB status in TBE. The associations of *TNFRSF1A* and *TNF* variants with MIF and TNFα expression show that the activity of the mediators influencing BBB function may indeed associate with the genetic background. However, they did not translate into associations with Q_alb_. Of note, our pre-planned genetic association analysis involved SNPs in the genes for a few selected factors only, and not the ones that appeared the most strongly associated with BBB impairment in our multivariate regression models. Thus, further genotyping studies are warranted to elucidate the possible genetic background of differences in BBB permeability, concentrating on variability in additional loci, for example in genes associated with the activity of IL-12, IL-15, type III interferons, MMPs or adhesion molecules.

## 4. Materials and Methods

### 4.1. Patient Recruitment

Two hundred forty-one adult patients hospitalized in the Department of the Infectious Diseases and Neuroinfections of the Medical University in Białystok with the suspicion of viral meningitis or encephalitis during 2019–2023 were recruited into the research project “Factors influencing presentation and outcome of tick-borne encephalitis”. Tick-borne encephalitis was confirmed in 190 patients, who were subsequently studied simultaneously for multiple biochemical, cellular and genetic markers. The patients included in the TBE group (1) had a history of a tick bite or exposition to ticks in an endemic area within 3 weeks before the onset of symptoms, (2) presented with an acute febrile disease with symptoms suggestive of meningitis and/or encephalitis, (3) had a CSF pleocytosis ≥ 15/μL in a lumbar puncture on admission and (4) had specific anti-TBEV IgM antibodies detected in serum and/or in CSF on admission or seroconverted on follow-up, according to the European criteria for the confirmed TBE case [47]. Patients with unclear etiology of the CNS infection (for example with other CNS pathogens more likely than or probably co-existing with TBEV) or with co-existing severe infectious diseases (e.g., HIV-infected, with other acute viral or with purulent bacterial infections) were excluded. Depending on the presence of focal neurologic symptoms and/or altered mental status, patients were classified as having either uncomplicated meningitis (M, the milder presentation), meningoencephalitis or meningoencephalomyelitis (respectively, ME and MEM—the more severe presentation). The clinical severity was further assessed in a quantitative scale as described by Bogovič et al. [34].

The cohort of 100 patients analyzed here was selected based on the availability of (1) multiplex data on the concentrations of the soluble factors of interest from the paired CSF and serum obtained on admission day, (2) biochemical data to assess Q_alb_ during the same admission lumbar puncture and (3) recorded clinical data sufficient to assess the clinical severity in the Bogovič scale [34]. Eighty-nine of these patients, in whom the genotyping data were available, were subject to the analysis of the associations of the soluble factor concentrations with the genetic background. For the analysis of Q_alb_ genetic associations, we have used a larger group, including 94 patients from the main study cohort supplemented by retrospective data from 120 patients studied in the previous genotyping research program during 2016–2018. The additional patients were recruited in the same center with identical inclusion criteria, did not differ significantly from the main cohort in terms of demographics and clinical presentation, underwent the same DNA extraction and genotyping procedure and had Q_alb_ assessed with the same procedure in the same laboratory.

**Figure A4:**
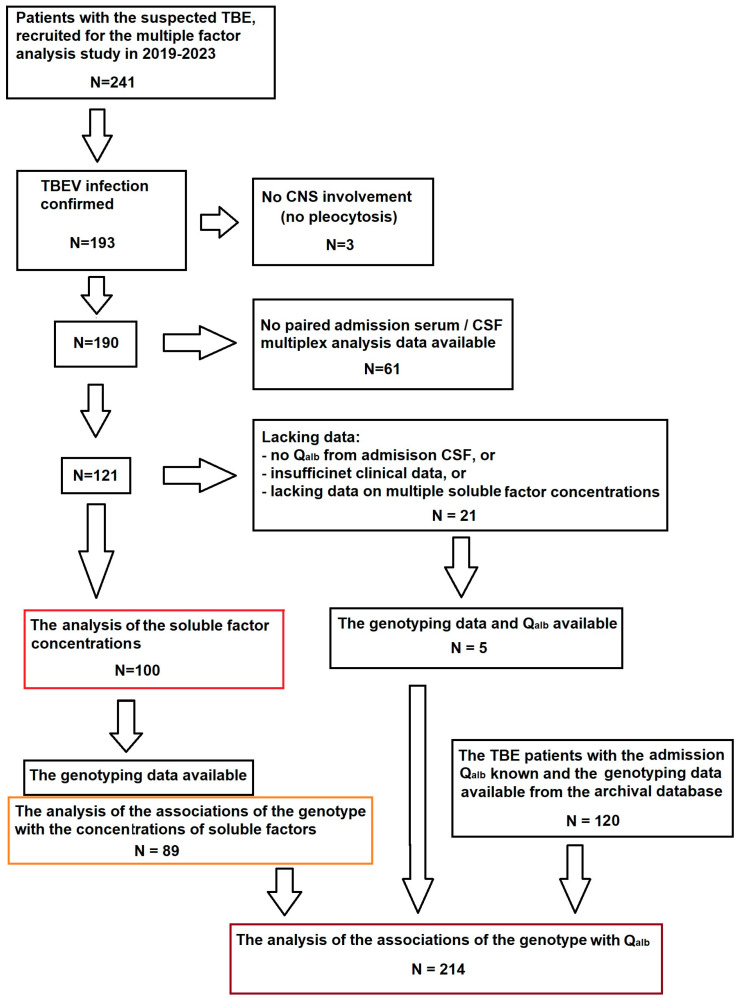
Flowchart of the patient recruitment into the study. The main study cohort (n = 100), highlighted in red, was selected from the multiple factor study group of TBE patients based on the availability of all the clinical and laboratory data relevant to the scope of the planned analysis. Of these patients, 89 had been genotyped and could be included in the sub-analysis of the soluble mediators associations with the genetic background, highlighted in orange. The group for the sub-analysis of Q_alb_ associations with the genetic background (n = 214), highlighted in brown, was supplemented with archived data from patients studied previously, recruited and analyzed with the same methodology in the same center.

**Figure A5:**
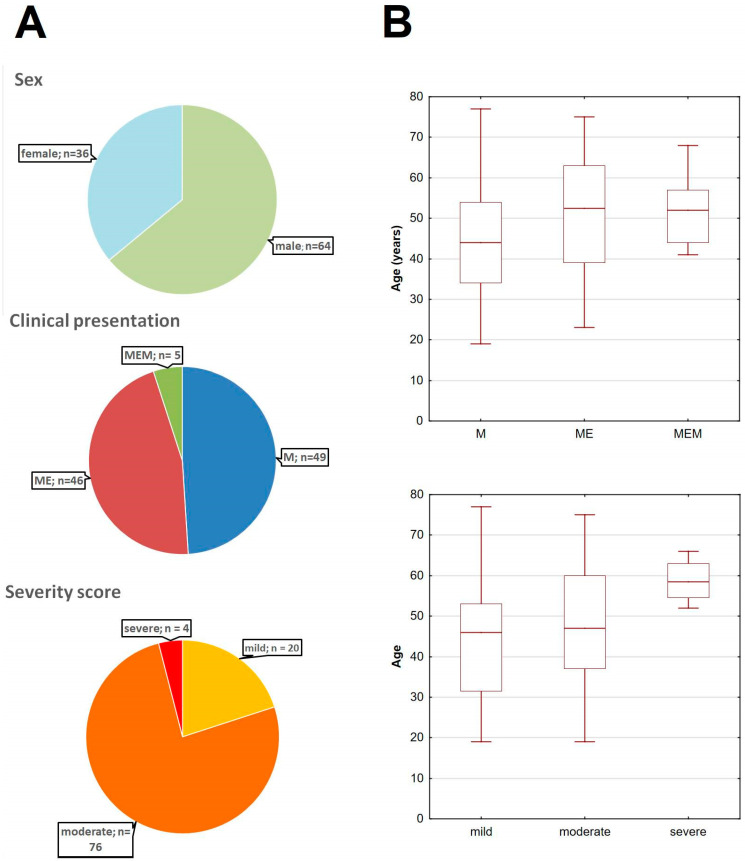
The basic demographic and clinical characteristics of the main study cohort of TBE patients. (**A**) The pie-charts of the cohort stratified dependent on sex (**top**), clinical presentation (**middle**) and the clinical severity according to the Bogovič score [34] (**bottom**). (**B**) The age of TBE patients stratified according to the clinical presentation (**top**) and severity score (**bottom**), showing the median (line), quartiles (box) and full range (whiskers); the tendency for a higher age in a more severe presentation and a higher severity score groups was non-significant (both *p* < 0.09); M—meningitis, ME—meningoencephalitis, MEM—meningoencephalomyelitis.

The flowchart of the patient selection scheme is shown in Figure A4.

The control groups were recruited separately and included (1) non-inflammatory CSF samples from hospitalized patients in whom CNS infection had been excluded by a lumbar puncture and (2) blood samples from healthy volunteers (blood donors).

All the patients and controls gave a written informed consent for the participation in this study.

### 4.2. Material and Basic Laboratory Examinations

The blood and CSF samples were obtained on admission to hospital, together with the samples for diagnostic examinations. This study was performed on serum samples obtained from 2.6 mL of blood collected on clot and centrifuged within an hour and on 1.0 mL CSF samples obtained on the same day. All the material was frozen on the day of collection, kept at −40 °C and thawed directly before use at the end of the study period.

The separate 1.4 mL sample was taken during a hospital stay for DNA isolation and genotyping.

Anti-TBEV IgM and IgG antibodies were detected with the Anti-TBE virus ELISA kits (EuroImmun, Lübeck, Germany) in serum and CSF samples obtained on admission, as well as on follow-up in originally seronegative patients, following the standard procedure.

The CSF pleocytosis, differential and albumin concentrations in serum and CSF were measured with standard laboratory techniques. The albumin quotient Q_alb_ was calculated from albumin concentrations in simultaneously obtained serum and CSF samples, as follows:

Q_alb_ = 1000 × (CSF_albumin_/serum_albumin_) (1)

The Q_alb_ values were considered elevated if >6.5 for patients ≤ 40 years old and >8.0 for those > 40 years old [48].

### 4.3. Micro-Bead Assay

The customized Luminex assays (R&D Systems, Minneapolis, MN, USA) were used for quantification of concentrations of 84 biomarkers in paired serum and CSF samples from TBE patients, as well as in non-inflammatory control serum and CSF samples. The factors analyzed included proinflammatory cytokines G-CSF, GM-CSF, MIF, TNFα, IL-1α, IL-1β, IL-2, IL-6, IL-12, IL-15, IL-16, IL-17A, IL-17E, IL-17F, IL-21, IL-22, IL-23 and IL-33; soluble receptors and receptor antagonists for selected of these cytokines (sIL-1RI, sIL-1RII, IL-1ra, sIL-6Rα, sTNFRI, sTNFRII); interferons IFNα_1_, IFNα_2_, IFNβ, IFNγ, IL-28A, IL-28B and IL-29; immunomodulatory/anti-inflammatory cytokines IL-10 and TGFβ; vascular endothelial growth factor (VEGF); matrix metalloproteinases MMP1, MMP2, MMP3, MMP7, MMP8, MMP9 and MMP12; soluble adhesion molecules: vascular cell adhesion molecule (sVCAM), platelet endothelial cells adhesion molecule (sPECAM) and intracellular adhesion molecule (sICAM); and E-, L- and P-selectins. Measurements were performed according to the manufacturer’s instruction on a Bio-Plex 200 System (Bio-Rad Laboratories, Hercules, CA, USA). The concentration of each biomarker was expressed in pg/mL and values below the lower detection limit were considered 0.

### 4.4. Genotyping

Genomic DNA was extracted from fresh blood samples with QIAamp DNA Blood Mini Kit (QIAgen, Hilden, Germany), re-suspended in 200 µL of AE buffer (QIAgen) and stored at 4 °C.

TaqMan SNP (Applied Biosystems/Life Technologies, Foster City, CA, USA) genotyping assays were performed following the manufacturer’s protocol and using a real-time PCR technique on the StepOne thermal cycler (Applied Biosystems/Life Technologies). Genotypes were identified with TaqMan Genotyper Software v1.0.1 (Applied Biosystems/Life Technologies). The following common single nucleotide polymorphisms (SNPs) were evaluated: rs3775291 and rs5743305 in *TLR3*, rs4986790 in *TLR4*, rs755622 in *MIF*, rs1800629 in *TNF*, rs767455 and rs4149570 in *TNFRSF1A*, rs1061622 and rs3379 in *TNFRSF1B*, rs1800896 and rs1800872 in *IL10*.

### 4.5. Data Analysis

The analyses were first performed with a pre-defined set of proinflammatory and immunomodulatory cytokines previously postulated to influence BBB function in *Flavivirus* encephalitis and belonging to the MIF-TNFα–dependent inflammatory signaling axis (MIF, TNFα, IL-1α, IL-1β, IL-6, IL-12), their natural modulators/antagonists (sIL-1RI, sIL-1RII, IL-1ra, sIL-6Rα, sTNFRI, sTNFRII, IL-10, TGFβ) as well as selected matrix metalloprotinases (MMP1, MMP2, MMP3, MMP7, MMP8, MMP9, MMP12). In a follow-up analysis, we also included VEGF, soluble adhesion molecules, selectins, interferons and a wider set of proinflammatory interleukins.

The data were analyzed with Statistica 13.0 software (TIBCO Software Inc., Palo Alto, CA, USA). We have tested for the upregulation of the studied mediators in TBE in comparison to controls with a Mann–Whitney U test and for their correlation with Q_alb_ with the Spearman rank test, with *p* < 0.05 considered significant. Following that, the multivariate regression models were built with Q_alb_ as a dependent variable and the concentrations of the soluble mediators in serum and CSF as independent variables. We avoided introducing variables with evidently non-linear association with Q_alb_ on visual inspection or strongly intercorrelated variables with a tolerance coefficient < 0.1. For that reason, we did not include the serum and CSF concentration of a same mediator in a single analysis, choosing one of these values based on the physiological relevance and the results of the initial analyses. In selected cases, we tested including ratios of the concentrations of the antagonistic mediators (for example IL-6Rα to IL-6) instead of pairs of individual concentrations as separate variables. The patients with P-selectin concentrations in CSF exceeding 1000 pg/mL (two cases) or MIF concentrations in serum exceeding 18,000 pg/mL (three cases) were excluded from analyses involving these parameters because of their clearly non-linear association with Q_alb_ in these value ranges. The selected variables were used as inputs to the step-wise regression analyses, with the resulting models tested and refined manually.

To identify genetic variability associated with BBB function, the Q_alb_ values and the selected cytokine concentrations were compared between patients with different genotypes in all the studied loci with non-parametric Kruskal–Wallis ANOVA.

## 5. Conclusions

We do not detect the evident correlation of BBB disruption with the severity of TBE, although there is a tendency for a more significant impairment in a small group of patients with the most severe disease.

The expression of general proinflammatory cytokines, especially IL-6 and TNFα, contributes to BBB disruption in TBE. The results are consistent with a BBB dysfunction being dependent more on the intrathecal then on the peripheral inflammation, as previously suggested by TBE animal models.

We identify novel soluble mediators associated with BBB function in TBE, including potentially disruptive IL-28A and potentially protective IL-12 and IL-15. The association of IL-12 and IL-15 with a better-preserved BBB may reflect their deeper involvement in the control of early stages of the TBEV infection.

Our results regarding the hypothesized protective role of type I interferons on the BBB are ambiguous.

We do not confirm a significant involvement of VEGF in BBB disruption in TBE.

We suggest that the dominant role in the BBB breakdown on the enzymatic level is played by the peripherally expressed MMP9, intrathecally expressed MMP7 and/or MMP3. The role of MMP8 alongside MMP9 in TBE is unclear.

The CSF concentrations of adhesion molecules L-selectin and sVCAM are the strong correlates of BBB disruption. This may reflect the leukocyte transmigration and its relation to BBB impairment.

We have not identified any genetic factors influencing BBB function in human TBE, but the scope of our study in that respect was limited to genes related to MIF, TNFα and IL-10 signaling. A further research is warranted, involving a more complete list of genetic polymorphisms.

## Figures and Tables

**Table 1:** The basic cerebrospinal fluid inflammatory parameters in the group of 100 patients with tick-borne encephalitis and in its subgroups defined based on clinical presentation and on severity.

	Pleocytosis (cells/μL)			Albumin (mg/dL)	Q_alb_
	Total	Lymphocytes	Neutrophils		
TBE (n = 100)	94.5 [57.5–160.0]	56.7 [32.4–80.0]	14.4 [7.4–32.9]	43.53 [34.18–55.08]	10.57 [8.44–13.85]
Stratified according to presentation:					
M (n = 49)	90.0 [60.0–185.0]	54.4 [33.2–102.0]	14.3 [8.4–24.0]	43.26 [32.03–54.34]	10.30 [8.19–12.56]
ME (n = 46)	94.0 [56.0–135.0]	55.0 [26.6–77.4]	14.6 [6.6–32.9]	45.65 [35.63–57.63]	10.85 [8.62–15.92]
MEM (n = 5)	157.5 [114.0–340.0]	83.3 [53.4–116.1]	29.4 [9.1–246.2]	53.36 [40.84–56.79]	13.54 [8.84–16.56]
Stratified according to the severity in a quantitative scale ^a^:					
mild ^a^ (n = 20)	84.5 [55.0–129.0]	46.5 [25.1–85.3]	14.4 [8.1–32.9]	43.11 [35.91–50.44]	10.57 [8.74–11.64]
moderate ^a^ (n = 76)	95.5 [57.5–160.0]	59.1 [32.6–90.2]	13.9 [6.6–26.7]	44.85 [34.18–54.97]	10.52 [8.39–13.85]
severe ^a^ (n = 4)	130 [86.5–340.0]	55.0 [49.7–81.3]	37.9 [18.7–246.2]	51.71 [36.3–67.9]	12.61 [8.10–17.58]

Shown are the median and quartiles in square brackets: TBE—tick-borne encephalitis; M—meningitis; ME—meningoencephalitis; MEM—meningoencephalomyelitis; ^a^ as described in the Material and Methods, Section 4.1.

**Table 2:** Correlations between the cellular parameters of the cerebrospinal fluid and the albumin quotient value in the group of 100 patients with tick-borne encephalitis.

CSF Parameter	Correlation with Q_alb_^a^	Statistical Significance
total pleocytosis	0.281	*p* < 0.01
lymphocytes	0.250	*p* < 0.05
neutrophils	0.029	NS

CSF—cerebrospinal fluid; NS—non-significant; Q_alb_—albumin quotient (calculated as in the Material and Methods, Section 4.2.); ^a^—Spearman rank coefficient.

**Table 3:** The concentrations of the soluble mediators in serum and cerebrospinal fluid of patients with tick-borne encephalitis that were tested as independent variables in the models of cerebrospinal fluid albumin quotient, with their statistical interpretation.

Parameter	Number of TBE Samples	Median [Quartiles]	Increase Compared to Controls ^a^ *p*	Correlation with Q_alb_ R (*p*)
MIF in serum	94	2493.1 [1746.9–3856.8]	decreased; *p* < 10^−5^	NS
MIF in CSF	98	21,548 [14,487–30,721]	*p* < 10^−6^	R = 0.246 (*p* < 0.05)
TNFα in serum	98	4.08 [2.66–6.15]	decreased; *p* < 0.05	NS
TNFα in CSF	99	6.71 [4.82–9.57]	*p* < 10^−6^	R = 0.451 (*p* < 10^−5^)
TNF-RI in serum	96	1835.2 [1569.5–2172.2]	*p* < 10^−6^	R = 0.351 (*p* < 0.001)
TNF-RI in CSF	98	2270.6 [1770.0–2965.4]	*p* < 10^−5^	R = 0.444 (*p* < 10^−5^)
TNF-RII in serum	96	2713.3 [2080.6–3391.4]	NS	NS
TNF-RII in CSF	98	2284.3 [1781.7–3147.2]	*p* < 10^−6^	R = 0.494 (*p* < 10^−6^)
IL-1α in serum	95	0.46 [0.00–7.03]	decreased; *p* < 10^−6^	NS
IL-1α in CSF	95	16.59 [14.09–20.05]	*p* < 10^−6^	R = 0.399 (*p* < 10^−4^)
IL-1β in serum	98	0.26 [0.09–0.61]	decreased; *p* < 0.001	R = 0.256 (*p* < 0.05)
IL-1β in CSF	99	1.53 [0.84–4.06]	*p* < 10^−5^	R = 0.408 (*p* < 10^−4^)
IL-1ra in serum	95	781.57 [566.23–1137.0]	*p* < 10^−6^	R = 0.255 (*p* < 0.05)
IL-1ra in CSF	95	4621.3 [2665.6–8189.9]	*p* < 10^−6^	R = 0.423 (*p* < 10^−4^)
IL-1RI in serum	98	1571.6 [1333.7–1829.9]	NS	NS
IL-1RI in CSF	99	56.21 [45.19–71.40]	NS	R = 0.246 (*p* < 0.05)
IL-1RII in serum	96	6944.5 [5523.6–8004.7]	*p* = 0.098	R = 0.271 (*p* < 0.01)
IL-1RII in CSF	98	348.10 [250.63–469.24]	*p* < 10^−6^	R = 0.549 (*p* < 10^−6^)
IL-6 in serum	98	4.84 [2.70–7.57]	*p* < 10^−6^	R = 0.281 (*p* < 0.01)
IL-6 in CSF	99	341.70 [169.55–753.84]	*p* < 10^−6^	R = 0.327 (*p* < 0.001)
IL-6Rα in serum	96	30,060 [24,655–34,576]	decreased; *p* < 10^−5^	NS
IL-6Rα in CSF	98	2431.2 [1890.7–3192.8]	NS	NS
IL-10 in serum	95	79.32 [55.00–130.86]	*p* < 0.05	NS
IL-10 in CSF	95	471.79 [402.53–509.39]	*p* < 10^−6^	R = 0.392 (*p* < 10^−4^)
IL-12p70 in serum	95	2.39 [0.00–6.39]	decreased; *p* < 10^−5^	NS
IL-12p70 in CSF	95	17.39 [12.89–22.27]	*p* < 10^−6^	R = 0.332 (*p* < 0.01)
TGFβ in serum	95	82,104 [59,220–102,021]	*p* < 0.05	NS
TGFβ in CSF	97	4137.0 [3310.2–5236.3]	*p* < 10^−6^	R = 0.329 (*p* < 0.01)
MMP1 in serum	98	3430.8 [2064.1–5507.6]	*p* < 0.05	NS
MMP1 in CSF	99	0.00 [0.00–5.51]	*p* < 0.05	NS
MMP2 in serum	98	85,746 [65,726–99,819]	*p* < 0.05	NS
MMP2 in CSF	100	13,407 [11,303–15,135]	*p* < 0.001	R = 0.222 (*p* < 0.05)
MMP3 in serum	98	13,388 [9267.5–19,738]	NS	R = 0.206 (*p* < 0.05)
MMP3 in CSF	99	485.45 [344.55–697.10]	*p* < 0.001	R = 0.257 (*p* < 0.05)
MMP7 in serum	98	2954.1 [2270.1–3910.4]	decreased; *p* < 10^−4^	NS
MMP7 in CSF	99	0.00 [0.00–74.20]	NS	R = 0.221 (*p* < 0.05)
MMP8 in serum	96	8307.4 [4381.2–15,461.4]	*p* < 0.001	R = 0.364 (*p* < 0.001)
MMP8 in CSF	98	1501.8 [822.85–3337.0]	*p* < 10^−6^	R = 0.281 (*p* < 0.01)
MMP9 in serum	98	464,697 [329,169–644,743]	*p* < 10^−6^	R = 0.350 (*p* < 0.001)
MMP9 in CSF	100	617.82 [300.80–1144.7]	*p* < 10^−6^	NS
MMP12 in serum	98	38.44 [27.52–48.36]	decreased; *p* < 10^−6^	NS
MMP12 in CSF	99	0.00 [0.00–9.08]	decreased; *p* < 0.01	R = −0.299 (*p* < 0.01)
sVCAM-1 in serum	98	1,334,600 [894,706–1,792,300]	*p* < 10^−6^	NS
sVCAM-1 in CSF	99	458,393 [334,108–631,634]	*p* < 10^−6^	R = 0.475 (*p* < 10^−6^)
sPECAM-1 in serum	98	17,783 [12,262–22,083]	decreased; *p* < 10^−6^	NS
sPECAM-1 in CSF	99	0.00 [0.00–156.28]	*p* < 0.05	NS
sICAM-1 in serum	98	342,310 [260,654–579,716]	NS	R = 0.244 (*p* < 0.05)
sICAM-1 in CSF	99	15,426 [10,638–24,143]	*p* < 10^−5^	R = 0.419 (*p* < 10^−4^)
VEGF in serum	95	274.56 [146.17–393.13]	*p* < 0.05	NS
VEGF in CSF	95	50.67 [43.23–67.00]	*p* < 0.05	R = 0.229 (*p* < 0.05)
E-selectin in serum	98	25,107 [9632–37,008]	decreased; *p* < 0.01	NS
E-selectin in CSF	99	236.31 [167.91–352.17]	*p* < 10^−6^	R = 0.309 (*p* < 0.01)
L-selectin in serum	98	871,769 [708,576–1,011,200]	*p* < 0.001	NS
L-selectin in CSF	100	30,460 [21,733–46,597]	*p* < 10^−6^	R = 0.627 (*p* < 10^−6^)
P-selectin in serum	98	40,144 [33,664–47,361]	decreased; *p* < 10^−6^	NS
P-selectin in CSF	98	15.78 [0.00–110.32]	*p* < 0.01	R = 0.478 (*p* < 10^−6^)
IFNα_1_ in serum	96	0.00 [0.00–0.00]	NS	NS
IFNα_1_ in CSF	98	0.79 [0.00–2.13]	NS	R = 0.349 (*p* < 0.001)
IFNγ in serum	94	2.81 [1.77–4.54]	*p* < 10^−5^	R = 0.373 (*p* < 0.001)
IFNγ in CSF	95	86.10 [41.71–206.91]	*p* < 10^−6^	R = 0.327 (*p* < 0.01)
IL-28A in serum	95	354.47 [201.12–504.07]	decreased; *p* < 0.05	NS
IL28A in CSF	98	1562.9 [1431.5–1730.2]	*p* < 10^−5^	NS
IL28B in CSF	98	69.18 [0.00–107.31]	*p* < 0.01	R = 0.336 (*p* < 0.001)
G-CSF in serum	95	13.26 [3.80–25.52]	*p* < 0.001	NS
G-CSF in CSF	95	1094.3 [508.59–1870.7]	*p* < 10^−6^	0.310 (*p* < 0.01)
GM-CSF in CSF	99	0.23 [0.00–0.54]	*p* < 10^−4^	R = 0.374 (*p* < 0.001)
IL-2 in serum	95	0.00 [0.00–0.80]	decreased; *p* < 10^−6^	NS
IL-2 in CSF	95	3.95 [2.71–5.26]	*p* < 10^−6^	R = 0.295 (*p* < 0.01)
IL-15 in serum	97	2.78 [2.02–4.03]	*p* < 10^−6^	NS
IL-15 in CSF	99	9.81 [7.14–13.11]	*p* < 10^−5^	R = 0.328 (*p* < 0.001)
IL-16 in serum	95	197.28 [151.78–274.71]	*p* < 0.05	NS
IL16 in CSF	98	93.53 [57.76–132.98]	*p* < 10^−6^	R = 0.346 (*p* < 0.001)
IL17F in CSF	98	1.78 [0.00–5.06]	*p* < 0.001	R = 0.413 (*p* < 10^−4^)
IL-18 in serum	96	134.97 [103.77–174.89]	*p* < 0.05	NS
IL-18 in CSF	98	8.75 [6.47–12.01]	*p* < 10^−5^	R = 0.481 (*p* < 10^−5^)
IL-22 in CSF	88	9.58 [0.00–31.27]	*p* < 0.001	R = 0.444 (*p* < 10^−4^)
IL23 in CSF	98	822.48 [560.22–955.75]	*p* < 0.001	NS
IL33 in CSF	99	0.56 [0.38–0.77]	*p* < 0.001	R = 0.380 (*p* < 0.001)

TBE—tick-borne encephalitis; CSF—cerebrospinal fluid; ^a^—statistical significance as measured with U Mann–Whitney test; NS—non-significant; Q_alb_—cerebrospinal fluid albumin quotient; R—Spearman rank coefficient.

**Table 4:** The best multiple linear regression model of the cerebrospinal fluid albumin quotient in patients with tick-borne encephalitis, built with the full set of serum and cerebrospinal fluid concentrations of inflammatory, antiviral and immunomodulatory cytokines, their antagonists, adhesion molecules and matrix metalloproteinases.

N = 83	R = 0.90239172; R^2^ = 0.81431081; Adjusted R^2^ = 0.78554206; F = 28.305; *p* < 0.0000; SE of Estimate = 2.1536				
	β	β SE	b	b SE	*p*
Free parameter			5.541345	1.182264	<0.0001
L-selectin in CSF	0.470627	0.061473	0.000102	0.000013	<10^−6^
sVCAM-1 in CSF	0.310246	0.065830	0.000006	0.000001	<0.0001
MMP8 in serum	0.237331	0.055236	0.000130	0.000030	<0.0001
P-selectin in CSF	0.222327	0.059302	0.009041	0.002411	<0.001
IL-28A in serum	0.150432	0.058221	0.003485	0.001349	<0.05
MMP7 in CSF	0.126354	0.057924	0.012620	0.005785	<0.05
MIF in serum	0.040728	0.054889	0.000072	0.000097	*p* = 0.461
IL-15 in serum	−0.140665	0.059938	−0.383169	0.163271	<0.05
IL-6Rα/IL-6 in serum	−0.152763	0.056150	−0.000080	0.000029	<0.01
TNFRII/TNFα in CSF	−0.195770	0.054986	−0.003652	0.001026	<0.001
IL12p70 in serum	−0.279800	0.063017	−0.298105	0.067139	<0.0001

The model parameters are presented in the top row, with the parameters for individual independent variables below. The variables are ordered from the strongest positive to the strongest negative correlation. Patients with MIF concentration in serum >18,000 pg/mL and P-selectin concentration in CSF > 1000 pg/mL and two patients with multiple outlying values were excluded from the analysis. SE—standard error; CSF—cerebrospinal fluid.

## Data Availability

The datasets used and/or analyzed during the current study are available from the corresponding author on reasonable request.

## Appendix A

The illustration of the strong intercorrelation between MMP8 and MMP9 concentration in the serum and CSF of patients with tick-borne encephalitis.

## Appendix B

The additional data on the results of the multivariate linear regression analysis, as referenced in the Results Section 2.3.2 and Section 2.3.4.

**Table A1:** The best multivariate linear regression model of the cerebrospinal fluid albumin quotient in patients with tick-borne encephalitis, built with the serum and cerebrospinal fluid concentrations of the MIF signaling pathway proinflammatory cytokines, their antagonists and matrix metalloproteinases.

N = 96	R = 0.60666832; R^2^= 0.36804645; Adjusted R^2^= 0.34026827; F = 13.249; *p* < 0.00000; SE of Estimate = 3.8735				
	β	β SE	b	b SE	*p*
Free parameter			1.857970	1.670239	0.269
TNFα in CSF	0.589526	0.100246	0.591526	0.100586	<10^−6^
IL1-RI in CSF	0.360412	0.089731	0.093910	0.023380	<0.001
MMP8 in serum	0.238308	0.084190	0.000097	0.000034	<0.01
MIF in CSF	−0.204854	0.098476	−0.000063	0.000030	<0.05

The model parameters are presented in the top row, with the parameters for individual independent variables below. The independent variables are ordered from the strongest positive to the strongest negative correlation. SE—standard error; CSF—cerebrospinal fluid.

**Table A2:** The multiple linear regression model of the cerebrospinal fluid albumin quotient in patients with tick-borne encephalitis, built as in Table 4 but without including the concentration ratios of proinflammatory cytokines and their soluble antagonists as independent variables.

N = 83	R= 0.88266882; R^2^ = 0.77910424; Adjusted R^2^ = 0.74842428; F = 25.395; *p* < 0.0000; SE of Estimate = 2.3356				
	β	β SE	b	b SE	*p*
Free parameter			3.095392	1.051678	<0.01
sVCAM-1 in CSF	0.362972	0.069781	0.000007	0.000001	<10^−5^
L-selectin in CSF	0.354030	0.067946	0.000077	0.000015	<10^−5^
P-selectin in CSF	0.221643	0.064324	0.008976	0.002605	<0.001
MMP8 in serum	0.193163	0.060886	0.000106	0.000033	<0.01
IL-28A in serum	0.171075	0.062259	0.003968	0.001444	<0.01
IFNγ in serum	0.166918	0.069668	0.209407	0.087402	<0.05
MMP7 in CSF	0.162504	0.059987	0.014411	0.005320	<0.01
MIF in serum	0.077664	0.058618	0.000137	0.000103	=0.189
IL-15 in serum	−0.174043	0.068555	−0.479573	0.188902	<0.05
IL12p70 in serum	−0.262003	0.065528	−0.274700	0.068703	<0.001

The model parameters are presented in the top row, with the parameters for individual independent variables below. The variables are ordered from the strongest positive to the strongest negative correlation. Patients with MIF concentration in serum >18,000 pg/mL. P-selectin concentration in CSF > 1000 pg/mL and two patients with multiple outlying values were excluded from the analysis. SE—standard error; CSF—cerebrospinal fluid.

## Appendix C

The summary of the results of the genotyping of the selected SNPs in the study TBE group and in the enlarged cohort of TBE patients.

**Table A3:** The distribution of the genotypes in the analyzed loci in the tick-borne encephalitis patient cohort.

Gene	Polymorphism	Genotype	Frequency [n (%)]	
			in a Full Cohort (n = 214)	in a Soluble Mediator Study Group (n = 89)
*TLR3*	rs3775291	C/C T/C T/T	93 (43.5%) 108 (50.5%) 13 (6.1%)	40 (44.9%) 48 (53.9%) 1 (1.1%)
	rs5743305	A/A T/A T/T	32 (15.0%) 85 (39.7%) 97 (45.3%)	10 (11.2%) 39 (43.8%) 40 (44.9%)
*TLR4*	rs4986790	A/A A/G G/G	184 (86.0%) 29 (13.6%) 1 (0.5%)	74 (65.9%) 15 (16.9%) 0 (0.0%)
*MIF*	rs755622	G/G G/C C/C	137 (64.0%) 72 (33.6%) 5 (2.3%)	60 (67.4%) 28 (31.5%) 1 (1.1%)
*TNF*	rs1800629	G/G A/G A/A	173 (80.8%) 36 (16.8%) 5 (2.3%)	73 (82.0%) 12 (13.5%) 4 (4.5%)
*TNFRSF1A*	rs767455	C/C C/T T/T	53 (24.8%) 118 (55.1%) 43 (20.1%)	23 (25.9%) 48 (53.9%) 18 (20.0%)
	rs4149570	C/C A/C A/A	86 (40.2%) 102 (47.7%) 26 (12.1%)	41 (46.1%) 36 (40.4%) 12 (13.5%)
*TNFRSF1B*	rs1061622	T/T G/T G/G	134 (62.6%) 66 (32.8%) 14 (6.6%)	51 (57.3%) 26 (29.2%) 8 (9.0%)
	rs3397	T/T T/C C/C	73 (34.1%) 109 (50.9%) 32 (15.0%)	29 (32.6%) 46 (51.7%) 14 (15.7%)
*IL10*	rs1800896	T/T T/C C/C	68 (31.8%) 105 (49.1%) 41 (19.2%)	26 (29.2%) 41 (46.1%) 22 (19.6%)
	rs1800872	G/G T/G T/T	119 (55.6%) 85 (39.7%) 10 (4.7%)	56 (62.9%) 29 (32.6%) 4 (4.5%)

**Table A4:** The value of albumin quotient (Q_alb_) stratified according to the genotypes in the analyzed loci in a cohort of 214 patients with tick-borne encephalitis. There are no statistically significant differences between the genotypes.

Gene	Polymorphism	Genotype	Q_alb_ Median (Quartiles)
*TLR3*	rs3775291	C/C T/C T/T	10.85 (8.37–13.99) 10.17 (7.54–13.46) 10.17 (7.54–14.15)
	rs5743305	A/A T/A T/T	10.67 (7.52–11.56) 11.28 (8.23–13.99) 10.85 (7.93–14.46)
*TLR4*	rs4986790	A/A A/G G/G	10.52 (7.93–13.81) 10.64 (8.66–16.65) NC
*MIF*	rs755622	G/G G/C C/C	10.22 (7.77–14.79) 10.91 (8.08–13.39) 10.60 (10.00–12.25)
*TNF*	rs1800629	G/G A/G A/A	10.71 (7.77–14.31) 10.40 (8.55–12.10) 9.55 (7.98–11.98)
*TNFRSF1A*	rs767455	C/C C/T T/T	10.67 (8.52–14.31) 9.84 (7.50–13.43) 11.61 (8.87–14.83)
	rs4149570	C/C A/C A/A	10.27 (8.42–13.99) 10.84 (7.61–13.40) 11.95 (8.37–14.47)
*TNFRSF1B*	rs1061622	T/T G/T G/G	10.77 (8.19–14.47) 10.40 (7.67–13.36) 10.68 (7.93–13.87)
	rs3397	T/T T/C C/C	10.50 (7.52–14.80) 10.85 (8.39–13.91) 9.51 (7.99–12.28)
*IL10*	rs1800896	T/T T/C C/C	10.85 (7.61–14.73) 9.86 (7.99–12.91) 10.84 (8.24–13.46)
	rs1800872	G/G T/G T/T	10.84 (8.24–13.99) 9.68 (7.45–12.98) 14.73 (10.85–18.07)

NC—non-calculable because of the small case number.

## Appendix D

The flowchart and characterization of the study cohort.
